# Fatigue self-management led by occupational therapists and/or physiotherapists for chronic conditions: A systematic review and meta-analysis

**DOI:** 10.1177/17423953211039783

**Published:** 2021-09-13

**Authors:** Sungha Kim, Ying Xu, Kelly Dore, Rebecca Gewurtz, Nadine Larivière, Lori Letts

**Affiliations:** 1 63662 School of Rehabilitation Science, 3710 McMaster University, Hamilton, ON, Canada; 2 Department of Medicine, 12362 McMaster Education Research, Innovation & Theory (MERIT), 12370 David Braley Health Science Centre, Hamilton, ON, Canada

**Keywords:** Fatigue, self-management, occupational therapy, physiotherapy, chronic conditions

## Abstract

**Objective:**

The aim of this study was to investigate the effectiveness of occupational therapist-/physiotherapist-guided fatigue self-management for individuals with chronic conditions.

**Methods:**

Eight databases, including MEDLINE and EMBASE, were searched until September 2019 to identify relevant studies. Randomised controlled trials and quasi-experimental studies of self-management interventions specifically developed or delivered by occupational therapists/physiotherapists to improve fatigue symptoms of individuals with chronic conditions were included. A narrative synthesis and meta-analysis were conducted to determine the effectiveness of fatigue self-management.

**Results:**

Thirty-eight studies were included, and fatigue self-management approaches led by occupational therapists/physiotherapists were divided into six categories based on the intervention focus: exercise, energy conservation, multimodal programmes, activity pacing, cognitive-behavioural therapy, and comprehensive fatigue management. While all exercise programmes reported significant improvement in fatigue, other categories showed both significant improvement and no improvement in fatigue. Meta-analysis yielded a standardised mean difference of the overall 13 studies: 0.42 (95% confidence interval:−0.62 to − 0.21); standardised mean difference of the seven exercise studies was −0.55 (95% confidence interval: −0.78 to −0.31).

**Discussion:**

Physical exercises inspired by the self-management principles may have positive impacts on fatigue symptoms, quality of life, and other functional abilities.

## Introduction

With increasing life expectancy, people are likely to experience chronic health conditions. Chronic health conditions share characteristics: persistency, irreversible pathological changes, possibility of remaining disabilities, and the need for rehabilitation services.^1^ Living with chronic conditions may lead individuals to cope with a variety of symptoms consistently, and fatigue is a common and disabling symptom of chronic conditions.^2,3^

Fatigue is a subjective experience characterised by decreased energy levels, a need for more rest, or any activity level changes in recent days;^2,4^ these patterns are similar across chronic conditions.^2^ Persons with chronic heart failure, multiple sclerosis (MS), cancer, and lupus report prevalence rates of fatigue ranging from 50% to 90%.^5–8^ Individuals with other chronic conditions, such as arthritis, asthma, emphysema, and anemia, are at a higher risk of experiencing fatigue than people without chronic conditions (odds ratio: 1.9–2.9).^9^ Fatigue affects physical function, role accomplishment, and relationships with others;^10^ it is considered the main factor that decreases the quality of life in individuals with MS.^8^ Although few available treatments exist specifically for fatigue,^3^ self-management principles are often applied to encourage individuals with chronic conditions to actively manage their fatigue symptoms.^11^

Chronic health conditions cannot be cured but can be managed, and this emphasises the role of the individual over the course of chronic diseases.^12–16^ Self-management principles assume that individuals living with a condition are experts who can obtain information regarding their health issues and take steps to manage their own condition.^17^ Health education is a core element in the chronic conditions’ management because it increases individuals’ ability to effectively self-manage their conditions.^18^ Self-management of chronic conditions can vary from monitoring the health condition to being knowledgeable about medical devices or tools needed on a regular basis. Self-management can also include taking medicine at appropriate intervals or adapting one’s lifestyle to reduce chronic symptoms.^19^

Symptom-specific self-management may be more practical and beneficial than more generic self-management strategies. However, most self-management approaches consist of multiple components aiming to promote overall health among people with chronic conditions.^17^ It might be difficult to determine which component is most relevant to improving fatigue. Thus, evidence of self-management specifically designed to improve fatigue may help clinicians understand how to help people with chronic conditions effectively deal with fatigue.

Self-management is used in rehabilitation to encourage people with chronic conditions to take steps to prevent their symptoms from worsening.^19^ Among rehabilitation health professionals, the role of occupational therapists (OTs) and physiotherapists (PTs) in self-management is of particular interest because they spend substantial time with their clients during the rehabilitation process, an important period for learning self-management strategies.^20^ Physiotherapy and occupational therapy practices emphasise active client involvement in building knowledge related to clients’ health conditions.^21^ However, the OTs and PTs’ roles in supporting fatigue self-management among people living with chronic health conditions have not been systematically reviewed. There is a need to better understand the evidence developed or delivered by OTs and/or OTs of fatigue self-management for people with chronic conditions.

The systematic review and meta-analysis aim to investigate the effectiveness of OT- and/or PT-informed fatigue self-management programmes for individuals with chronic conditions. The guiding research question is, “What is the effectiveness of fatigue self-management for people with chronic conditions developed and/or delivered by OTs and/or PTs?”

## Methods

This systematic review and meta-analysis (registered on PROSPERO #CRD42018090244) were conducted in accordance with the Preferred Reporting Items for Systematic Reviews and Meta-Analyses (PRISMA) guidelines.^22^ We adhered to the following processes to conduct a rigorous systematic review: (1) identification of an explicitly defined, clinically relevant question; (2) development of a protocol with well-defined inclusion and exclusion criteria; (3) a systematic literature search of multiple databases; (4) thorough study identification; (5) systematic data abstraction; (6) risk-of-bias assessment; and (7) synthesis, summary, and presentation of findings.^23,24^

### Operational definitions

*Fatigue.* In this review, fatigue was considered secondary to chronic conditions and differentiated from general tiredness in that it is not alleviated by rest and sleep and is defined as unusual, extreme, persistent, or problematic physical/mental tiredness and lack of energy.^25^

*Self-management. Self*-management is defined as ‘the ability of the individual, in conjunction with family, community, and healthcare professionals, to manage symptoms, treatments, lifestyle changes, and psychosocial, cultural, and spiritual consequences of chronic diseases’ (p. 257).^26^ The common self-management processes include (1) setting goals; (2) conducting self-monitoring and reflective thinking; (3) making decisions; (4) planning for and engaging in specific health behaviours; (5) self-evaluating; and (6) managing physical, emotional, and cognitive responses in relation to health behaviour change.^27–29^

### Eligibility

Studies were either randomised controlled trials (RCTs) or non-RCTs, with at least one comparison group included.^30^ Studies were included if the intervention was a self-management approach specifically designed to improve fatigue experienced by individuals with at least one chronic condition and was developed and/or delivered by OTs and/or PTs.

We did not restrict by study outcomes, geography, or publication year, but only studies published in English were included. Studies were excluded if less than 50% of the intervention was about fatigue and if less than 50% was developed and/or delivered by OTs and/or PTs.

There has been debate about whether cancer should be viewed as a chronic condition. However, cancer is considered a chronic disease regardless of the cancer survivor’s disease status.^31^ Cancer survivors often need to deal with their remaining physical and psychosocial issues resulting from cancer or treatment,^32,33^ which prevent them from participating in daily activities and adversely affect their quality of life.^33^ Because of the nature of living with chronic conditions, cancer is on the World Health Organization’s list of chronic diseases.^34^ Thus, we considered cancer a chronic condition in this review. However, we excluded a study if participants with cancer were receiving active chemotherapy or radiation therapy during the trial to differentiate fatigue caused by chronic conditions from fatigue as a side effect of treatment.

### Literature search

After consulting a research librarian and piloting search strategies, MEDLINE, EMBASE, CINAHL, PubMed, Cochrane, OTseeker, PEDro, and Ageline were accessed. The initial searching was conducted until November 2017 and was updated until December 2020. The applied search terms were fatigue, chronic conditions, and OT/PT; an example search strategy in MEDLINE is presented in Supplementary Table 1. We also searched included studies’ reference lists to ensure the inclusion of all relevant studies.

### Study selection

Two reviewers (initials of the authors removed for the blind review process) independently screened titles and abstracts of identified studies and excluded any irrelevant studies, such as editorial papers and papers where fatigue was not the focus. The inclusion decision was made through full-text reviews conducted by two reviewers. Conflicts were resolved through discussion until both reviewers agreed to include or exclude studies. The online platform Covidence was used to screen the studies, keep a record of decisions, and resolve conflicts.

### Data extraction

Two reviewers extracted data on demographics, inclusion and exclusion criteria for participants, intervention protocols, and study outcomes. Only primary outcomes of each study were included in the analysis to ensure the focus of the intervention. If fatigue was included in the secondary outcome, it was also included in the analysis given the focus of fatigue self-management. When pilot studies assessed their interventions’ feasibility and reported fatigue as a secondary outcome, we only extracted the fatigue outcome data.

### Risk-of-bias appraisal tool

The Cochrane risk-of-bias tool^35^ was used to evaluate the internal validity of each included RCT study. The Risk of Bias in Non-randomised Studies – of Interventions (ROBINS-I) tool^36^ was used for non-RCTs. The conflict between the reviewers was resolved through discussion.

### Data synthesis and analysis

A narrative synthesis of the findings was conducted to narratively report the primary outcomes’ and fatigue outcome’s effectiveness compared to that of the comparison groups.

Meta-analysis of fatigue outcome was performed using RevMan 5.3, when appropriate. Because different outcome measures were used in the included studies, we calculated the standardised mean differences (SMDs: 95% confidence interval [CI]) and measures of heterogeneity with the χ^2^ and*I*^2^ statistics. We applied fixed or random-effect methods based on the magnitude of heterogeneity. The random-effect method was used if *I*^2^ statistics was ≥50%, meaning substantial or considerable heterogeneity;^35^ otherwise, a fixed-effect method was used.

### Certainty of evidence

After conducting meta-analysis, we evaluated the certainty of evidence and strength of recommendations for the fatigue outcome using the Grading of Recommendations, Assessment, Development and Evaluation (GRADE) system.^37^

## Results

### Study selection

**Figure 1.**
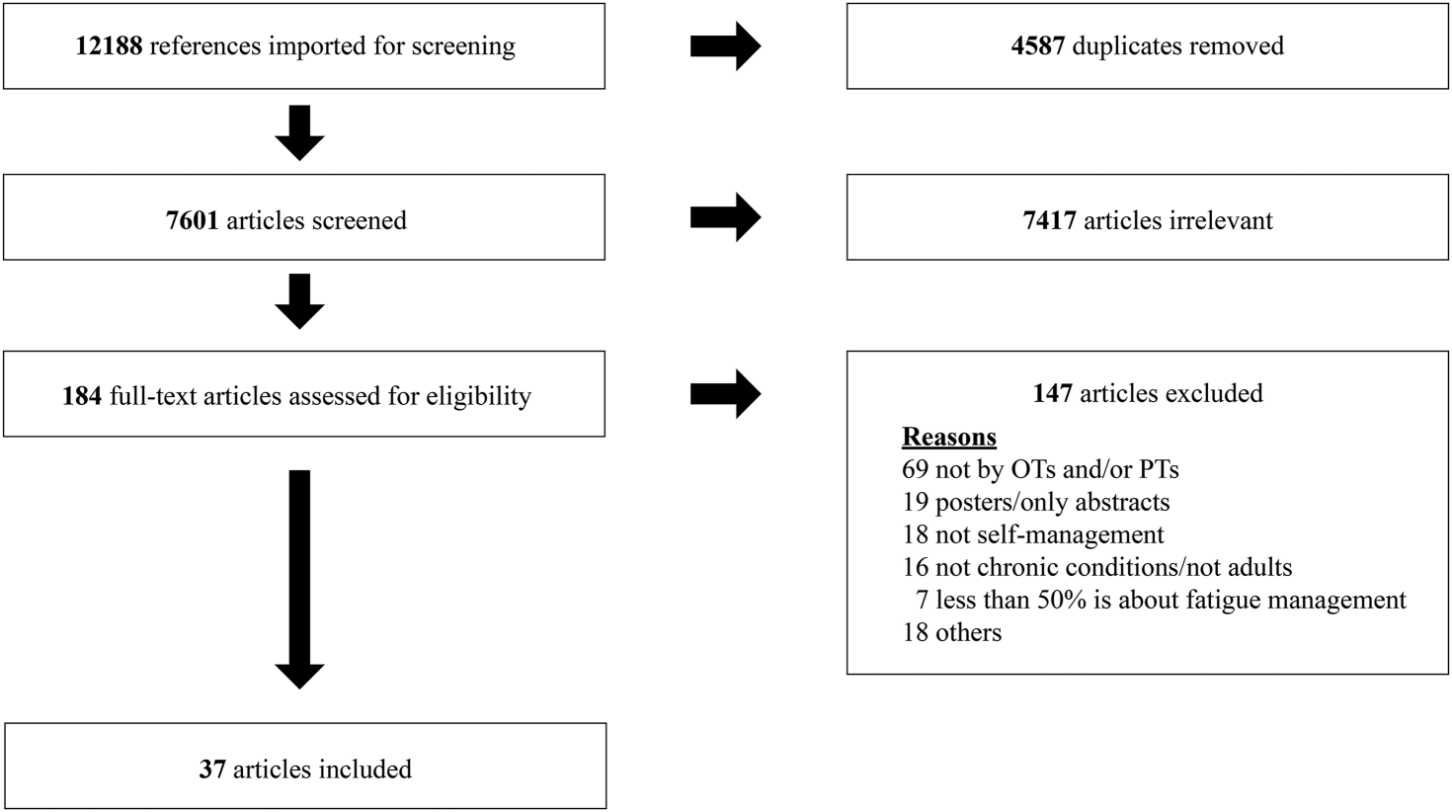
Preferred Reporting Items for Systematic Reviews and Meta-Analyses (PRISMA) flowchart of the study selection process.

Initially, 34 eligible articles (32 studies) were identified after screening 5918 articles; the observed agreementimp was 96% and the kappa inter-rater agreement was 0.5, which is considered ‘moderate’.^38^ Three studies were added after screening 1683 articles in September 2019, and one study was included after screening 778 studies in December 2020. The process of study selection was described by applying the PRISMA flowchart (Figure 1).

*Study characteristics*. Studies conducted from 1987 to 2019 in different countries were included in this systematic review and meta-analysis: the United States (8), the United Kingdom (7), the Netherlands (8), Ireland (2), Norway (2), Australia (1), Canada (1), Saudi Arabia (1), Spain (1), Sweden (1), Switzerland (1), and Turkey (1). Of the 36 studies included, 34 were RCTs and 2 used quasi-experimental designs. There were four crossover RCTs and one crossover non-RCT, and six 3-armed studies and one 4-armed study.

*Participant characteristics.* A total of 3109 participants were included from 36 primary trials. The chronic conditions varied, including chronic fatigue syndrome (CFS; *n* = 1190),^39–45^ MS (*n* = 1128),^2,46–59^ rheumatoid arthritis (RA; *n* = 611),^60–64^ cancer (*n* = 502),^65–69^ stroke (*n* = 83),^70^ type 2 diabetes with obesity (*n* = 80),^71^ chronic obstructive pulmonary disease (*n* = 65),^72^ facioscapulohumeral muscular dystrophy (*n* = 57),^73^ neuromuscular disease (*n* = 53),^74^ osteoarthritis (OA; *n* = 32),^75^ post-polio syndrome (*n* = 13),^2^ and Parkinson’s disease (*n* = 8).^2^ More detailed characteristics of the study participants, including age, disease, and duration, are provided in Supplementary Table 2.

### Assessment of risk of bias

**Figure 2.**
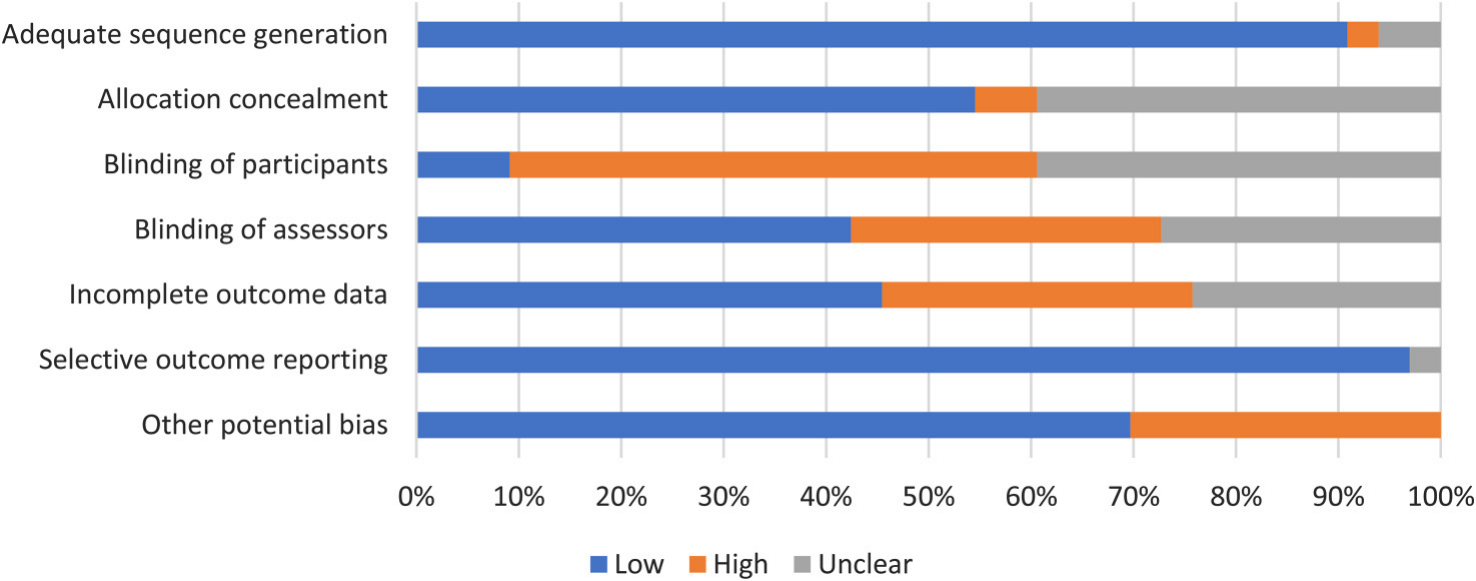
Risk-of-bias graph of randomised controlled trials (RCTs) describing ‘low’, ‘high’, and ‘unclear’ proportion.

Overall, included RCTs presented with high or unclear risk-of-bias for one or more domains. More than 90% of the included studies presented a low risk-of-bias in explaining the allocation sequence method in detail and in selective outcome reporting. However, only 3 of 34 RCTs blinded participants and less than 50% of the included studies blinded the trials’ assessors. More than 50% of the studies did not state the reasons for drop-outs or reported dissimilar reasons for drop-outs between groups, resulting in a high risk-of-bias in the incomplete outcome data domain. The risk-of-bias graph of RCTs (*n* = 34) is depicted in Figure 2.

Two quasi-experimental studies presented a serious or critical overall risk-of-bias due to a lack of information regarding dealing with missing data and blinding outcome assessors. A risk-of-bias of summary for each study is presented in Supplementary Table 3.

### Interventions

Fatigue self-management programmes were applied in different ways within the included studies. We categorised OT-/PT-led fatigue self-management approaches based on the focus of the intervention used to improve fatigue symptoms. This led to the following six categories: 12 exercise programmes,^39,44,49,54,60,61,65,67,69,71–73^ 10 energy conservation programmes (ECPs),^2,46–48,51–53,57,58,62^ seven multimodal programmes,^40,55,59,66,68,70,74^ three activity pacing programmes;^41,44,75^ three cognitive-behavioural therapy (CBT) programmes;^45,63,64^ and three comprehensive fatigue management programmes.^42,50,56^ Although some overlapping comprehensive fatigue management and energy conservation content was found, we decided to differentiate them because comprehensive fatigue management includes broader topics, including the importance of exercise,^42,50^ stress management,^56^ and sleep hygiene.^42,56^ Multimodal programmes combine specific types of interventions, such as an exercise programme combined with a CBT programme, but are not considered comprehensive. Among 36 trials, 15 were developed or delivered by PTs, 17 by OTs, and 4 studies by both OTs and PTs.

*Exercise programmes*. Different exercise interventions were identified: aerobic exercise,^49,54,67,69,71–73^ graded exercise,^39,44^ comprehensive home-based exercise,^60^ resistance exercise,^69^ person-centered physical therapy,^61^ and ambulant activity feedback.^65^ The exercise recommendations’ frequency, duration, and intensity varied across studies (Supplementary Table 2). The most common frequency of exercise was three times per week, but the time per session varied. The duration of included exercise studies ranged from 8 to 36 weeks. The intensity of exercise also varied, but low to moderate intensity was most commonly recommended.

Although most of the exercise interventions did not explicitly state that the interventions were based on a self-management approach, we considered the intervention to be a self-management one if the treatment applied at least one process associated with self-management in the literature,^27–29^ including (1) setting treatment goals, (2) monitoring and reflecting on performance progress, (3) participating in decision-making, (4) planning for and engaging in specific behaviours, and (5) self-evaluating and managing responses related to health behaviour change.^27–29^

*Energy conservation.* Seven out of the 10 energy conservation studies were designed using Packer’s programme.^76^ The programmes consisted of the following common topics: information on fatigue, importance of rest, balanced schedules, communication, priorities, activity analysis, and ergonomics.^2,46–48,51,53,57^ Eight ECPs were delivered in-person, while the other two were delivered remotely online^2^ or by teleconference.^47^

Interventions lasted from 3 to 12 weeks for approximately 2 hours per session.

*Multimodal programme.* Seven studies applied a multimodal approach: two studies^40,70^ combined cognitive therapy and graded activity training; one combined physical exercise and myofascial release massage;^66^ one combined physical activity and comprehensive fatigue self-management intervention;^59^ and another study combined aerobic exercise, exercise education, energy conservation education, and implementation and relapse prevention.^74^ A multidisciplinary rehabilitation programme^55^ including physical therapy, occupational therapy, and social work and a combination of physical therapy and CBT^68^ were included. Interventions mostly lasted 12–16 weeks, and their frequency varied from once a week for 2 hours to three times a week for 1.5 hours. Detailed intervention protocols are described in Supplementary Table 2.

*Activity pacing*. The three activity pacing programmes’ focus was on encouraging participants to pre-plan activities and take frequent breaks before symptom exacerbation.^41,44,75^ Two programmes’ duration was relatively short, ranging from 2 to 3 weeks for 1.5–4.5 hours in total;^41,75^ one programme lasted 36 weeks and involved up to 15 sessions.^44^

*CBT.* Three studies used CBT principles to promote fatigue self-management. Stubhaug et al.^45^ applied comprehensive CBT, including a psychiatrist and psychiatric nurse-led CBT and a PT-led body awareness therapy. The body awareness therapy focused on body sensations, breathing, posture, balance, and muscular tension and also discussed how improved body awareness can help participants better control their physical and mental tension.^45^ The other two CBT programmes promoting participants’ behavioural change related to self-managing fatigue by facilitating problem-solving, goal-setting, self-monitoring of activity, and energy management.^63,64^ The intervention lasted from 6 to 12 weeks for 1.5–2 hours per session.

*Comprehensive fatigue management programmes.* Comprehensive fatigue management programmes consisted of various topics, including possible causes of fatigue,^42,50,56^ its impacts on life,^42,50,56^ communications and relationships with others,^42,50,56^ body mechanics,^42,50,56^ daily activity analysis,^42,50,56^ balanced lifestyle,^42,50,56^ importance of exercise,^42,50^ stress management,^56^ and sleep hygiene.^42,56^ All programmes were delivered in person, lasted from 6 to 8 weeks, and typically occurred weekly for 1–3 hours.

### Narrative synthesis: Primary outcomes and effectiveness of fatigue self-management programmes

Various outcome measures were used to determine fatigue self-management’s effectiveness on fatigue impact and severity, quality of life, and other functional changes. The most frequently used measures were the Fatigue Impact Scale (FIS; *n* = 12), Medical Outcomes Study Short-Form Health Survey-36 Items (SF-36; *n* = 9) for quality of life, Checklist Individual Strength-fatigue subscale (CIS-f; *n* = 7), Fatigue Severity Scale (FSS; *n* = 6), and Canadian Occupational Performance Measure (COPM; *n* = 3) for performance improvement. The included outcome measures presented adequate reliability and validity; FIS, CIS, FSS, and SF-36 reported good internal consistency (Cronbach’s alpha ranged from 0.74 to 0.96).^77,78^ COPM was also adequately different from other measures, indicating good discriminant validity.^79^ For SF-36, six studies used the physical functioning subscale only, one used six subscales, and two studies used all eight subscales. Different aspects of fatigue were measured using severity, impact, and multidimensional scales. Detailed information regarding the measures included in the trials is presented in Supplementary Table 2.

*Exercise.* Eleven of 12 studies that featured an exercise programme reported statistically significant improvement in fatigue symptoms (*p* < 0.0001– <0.05),,^39,44,49,54,60,61,65,67,71–73^ improved quality of life (*p* = 0.0005–0.006),^39,44,54^ and sleep quality (*p* = 0.04)^60^ compared with the control groups for the clients with varying chronic conditions. The exception was a three-armed RCT^69^ that examined the effectiveness of aerobic exercise, resistance exercise, and usual care in people with breast cancer. The significance of between-group differences was not reported, but participants in the aerobic exercise programme experienced improved fatigue (*p* = 0.006), and participants in the resistance exercise programme improved functional capacity (*p* = 0.009) after the trial.

*ECPs.* In the nine studies that examining ECPs, there was no significant improvement (*p* = 0.58) in fatigue among participants with MS who participated in an ECP compared with those who only received information.^46^ Participants with RA who received an ECP did not experience significant improvement in fatigue compared with those who received traditional occupational therapy.^62^ Only one quasi-experimental study reported significant improvement in all three domains of FIS (cognitive, physical, and psychosocial) in the MS population.^57^ An RCT reported improvement only in the cognitive subscale (*p* = 0.001),^48^ and another RCT reported improved physical fatigue (*p* = 0.0002), social fatigue (*p* = 0.0005), and vitality (*p* < 0.0001) among individuals with MS.^53^ A teleconference-delivered fatigue management programme for clients with MS found participants’ improvement in fatigue impact (*p* = 0.0013 ∼ 0.0144) and physical roles (*p* = 0.0002) compared with the waitlist control group’s participants.^47^ Two studies of MS populations^51^ and chronic neurological conditions (MS, Parkinson’s disease, and post-polio syndrome)^2^ found no significant difference between the study group and the control group for fatigue and well-being outcomes. The inpatient energy management education programme did not report a significant improvement in fatigue compared with the progressive muscle relaxation control group at postintervention and follow-up (*p* > 0.05).^58^ Nevertheless, there was a statistically significant difference in self-efficacy at follow-up (p ≤ 0.05) and physical functioning–related quality of life at postintervention and follow-up in favour of the study group (*p* ≤ 0.05).^58^

*Multimodal programme.* Seven studies evaluated multimodal programmes. There was no statistically significant difference in fatigue levels for participants with stroke between the study group that received cognitive therapy combined with graded activity training and the control group that received cognitive therapy alone (*p* > 0.1).^70^ However, a better clinical improvement on fatigue was reported in the study group (58% vs. 24%).^70^ When compared with the waitlist group, an occupational therapy inpatient programme combining CBT and graded activity training reported improved fatigue (*p* < 0.05) and reduced emotional distress (*p* < 0.03).^40^ Incorporating myofascial release massage into the individual physical exercise intervention improved fatigue symptoms for individuals with breast cancer (*p *< 0.05).^66^ However, a multidisciplinary outpatient rehabilitation programme including physical therapy, occupational therapy, and social work did not present a significant improvement in fatigue (*p* = 0.39) compared with nurse consultation in the MS population.^55^ Compared with the waitlist group, the group that combined physical training and CBT showed significant improvement only in the physical fatigue subscale of Multidimensional Fatigue Inventory (*p* < 0.01). The group that received physical training alone improved in four of five domains (*p* < 0.001–<0.05).^68^ The group combining physical activity and comprehensive fatigue self-management presented a significant improvement in physical activity (*p* = 0.01) and fatigue (*p* = 0.03) compared with the contact-control intervention group; there was no significant difference in fatigue and physical activity outcomes between the physical activity only and physical activity plus comprehensive fatigue self-management intervention groups.^59^ A programme combining aerobic exercise training and education, energy conservation, and relapse prevention presented a statistically significant difference in COPM performance between the study group and the usual care control group, in favour of the former.^74^

*Activity pacing programmes.* Activity pacing programmes were evaluated in three studies. There was a significant difference in scores of COPM satisfaction with performance between the activity pacing self-management group and the relaxation group in individuals with CFS (*p* = 0.02).^41^ A tailored activity pacing intervention for people with OA showed significant improvement in fatigue interference compared with general pacing instruction (*p* = 0.02), but there was no difference in pain levels between the two groups (*p* = 0.35).^75^ Adaptive pacing therapy to help participants plan and pace their activity did not improve fatigue (*p* = 0.38) and physical function (*p* = 0.18) compared with specialised medical care alone in individuals with CFS.^44^

*CBT.* Three CBT programmes showed improved fatigue impact in people with RA (*p* < 0.001) compared with information-only or usual care,^63,64^ and improved fatigue severity (*p* = 0.014) and perception of illness severity (*p* = 0.001) in people with CFS compared with medication.^45^

*Comprehensive fatigue management programmes.* Comprehensive fatigue management programmes were evaluated in three studies. Individuals with MS saw improved fatigue impact compared to the control (waitlist) group (*p* = 0.008), but no significant difference in fatigue severity.^50^ A group-based fatigue management programme for clients with MS applied cognitive-behavioural and energy effectiveness techniques and found improved self-efficacy in controlling fatigue compared with the control group, local-practice-alone group, 1 month after the final session (*p* = 0.001).^56^ The self-efficacy of the study group’s participants remained significant 4 months after the final session (*p* = 0.48).^56^ Although this intervention did not significantly improve participants’ fatigue (*p* = 0.86) and quality of life (*p* = 0.46) at 1 month follow-up, study participants reported improved fatigue (*p* = 0.014) at 4-month follow-up.^56^ Participants with CFS who received usual care reported improved fatigue severity (*p* = 0.039) but no significant improvement in physical function (*p* = 0.21) compared with the group-based self-management programme.^42^

*Adverse events.* Of the 38 studies, it was unclear whether adverse events were measured in 23 studies. Nine studies reported no adverse events, and adverse events reported in six studies were not severe or not directly related to interventions. Adverse events were recorded by clients or observed by therapists during the intervention.

### Quantitative analysis: Meta-analysis of the fatigue outcome

Fatigue outcomes were used for the meta-analysis when mean and standard deviation data were available postintervention; we included median and interquartile ranges from one RCT^55^ by assuming that the outcome was similar to the normal distribution.^35^ Because of the heterogeneity of studies, we only conducted meta-analyses with studies where interventions were compared with a no-intervention or usual care control group, resulting in the inclusion of 13 RCTs for meta-analysis. We calculated the SMDs of the 13 fatigue self-management interventions. Among these 13 studies were two comprehensive fatigue management studies, two energy conservation studies, seven exercise studies, and two multimodal studies. Thus, we conducted a subgroup analysis with only seven exercise studies.

**Figure 3.**
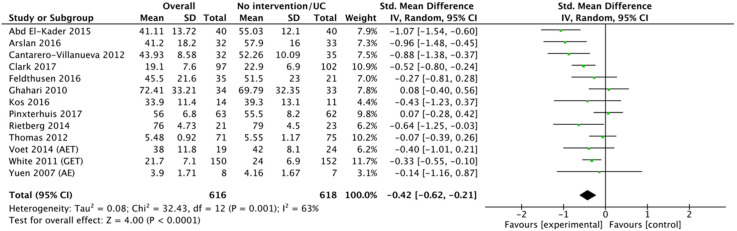
Standardised mean difference between post-overall fatigue self-management interventions and post-no intervention or usual care.

*Overall interventions.* Because of heterogeneity among studies (*I*^2 ^= 63%, *p* = 0.001), random-effect methods were applied. The SMDs (95% CI) of the 13 OT-/PT-led fatigue self-management studies compared with the no-intervention or usual care studies were − 0.42 (95% CI: − 0.62 to −0.21; see Figure 3). According to GRADE, the certainty of evidence was low mainly because of a serious risk of bias and heterogeneity across studies. Further research is likely to change our confidence in the estimate of the effect.^37^

**Figure 4.**
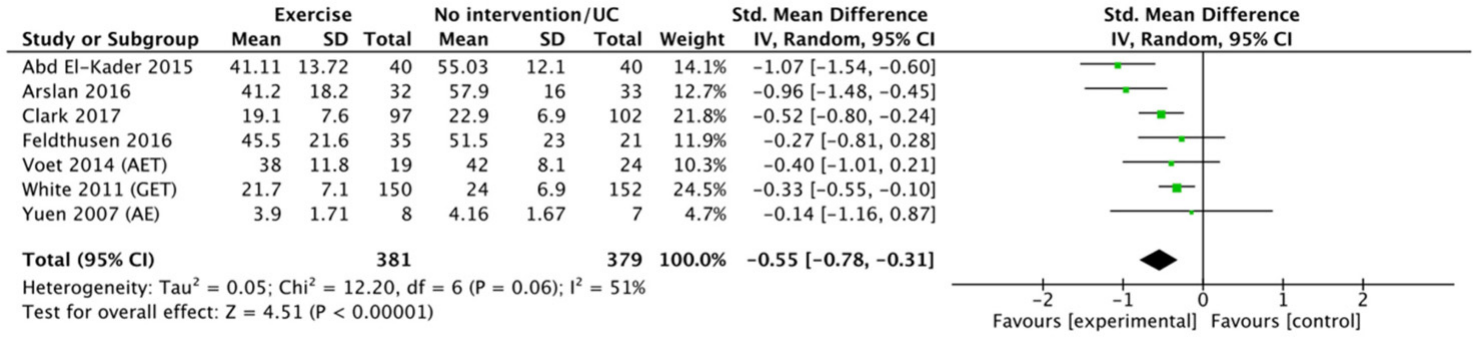
Standardised mean difference between post-exercise fatigue self-management interventions and post-no intervention or usual care.

*Exercise.* Because of heterogeneity among studies (*I*^2 ^= 51%, *p* < 0.00001), we applied the random-effect methods. The SMDs (95% CI) of exercise-focused fatigue self-management compared with no intervention or usual care were − 0.55 (95% CI: − 0.78 to − 0.31; Figure 4). The certainty of evidence on exercise fatigue management was also low because of a serious risk of bias and heterogeneity across the seven studies.

## Discussion

This systematic review and meta-analysis identified 38 articles (36 trials) addressing OT-/PT-led fatigue self-management strategies’ effectiveness in people with chronic conditions. The studies presented mixed results for the experimental groups in terms of fatigue self-management’s effectiveness on fatigue, quality of life, performance, self-efficacy, physical function, pain, and social participation compared with the control groups. There was heterogeneity across populations, intervention types and protocols, and outcomes, which may make it difficult for clinicians to decide whether they should use the findings from this review for individual clients.

The narrative synthesis presented inconsistencies in the available evidence; comprehensive fatigue management programmes, ECPs, activity pacing programmes, and multimodal programmes presented significant differences and no difference between the study and control groups in primary outcomes. One possible reason for these inconsistencies in the outcomes is that health behavioural changes occur over time.^76^ Lally et al.^80^ reported that it took a median time of 66 days to form a health-related habit (range: 18–254 days), which implies habit formation is a lengthy process. In our review, only one study^42^ conducted a 1-year follow-up evaluation; other follow-up intervals varied from 8 weeks to 6 months. This may suggest that longer follow-up times are necessary to evaluate the true impact of fatigue self-management programmes.

The results’ heterogeneity can be examined from another theoretical lens, the stages of change. According to Prochaska’s transtheoretical model of health behaviour change,^81^ there are six stages of change, which range from individuals having no intention to take action to the final stage, where people will no longer return to their former unhealthy habit. Thus, individuals who are less ready to adopt fatigue management strategies might take more time than people who are ready.

In addition, intervention duration may be a factor affecting outcomes. Longer intervention periods were related to positive effects.^82,83^ Most fatigue management programmes with inconsistent results included in our review ranged from 2 to 12 weeks in duration. These short durations might not be sufficient for positive effects on fatigue symptoms.

In our narrative synthesis, energy conservation presented mixed results in terms of effectiveness on fatigue, societal participation, quality of life, and self-efficacy. This result is similar to that of the systematic review of the effectiveness of the energy conservation intervention to reduce fatigue in individuals with MS, which reported limited evidence of the effectiveness of the energy conservation intervention group over the control group based on the best-evidence synthesis.^84^

Although fatigue itself is one reason for a lack of exercise or physical activity,^85^ the narrative synthesis of this review demonstrated positive results of exercise on fatigue, quality of life, and sleep quality, regardless of the exercise type. Furthermore, the meta-analysis presented a higher SMD compared to that in the overall fatigue management studies. Other reviews also reported the positive effect of exercise on fatigue among different chronic health conditions.^86–88^ However, our review is different from others regarding exercise’s effectiveness because our focus is on exercise programmes that incorporate self-management principles. Given the positive results of exercise self-management programmes, we suggest that exercise programmes that support participants’ active participation may also improve fatigue and other health-related outcomes.

Caution is needed, however, regarding exercise programmes’ provision. A systematic review of exercise’s effectiveness for the management of cancer-related fatigue found that further research was necessary to determine the most effective types of exercises to reduce fatigue.^87^ Only one three-armed RCT in our review examined the effectiveness of resistance exercise, aerobic exercise, and usual care control for breast cancer survivors; within-group differences showed that aerobic exercise improved fatigue symptoms, and resistance exercise improved functional capacity.^69^ Because this study did not report the statistical difference between the groups, we cannot determine whether these two exercise types are significantly different in their outcomes.^69^ In addition, it is difficult to recommend the most effective exercise duration and intensity because of variations in the exercise protocols included in our review. Thus, further research is needed to determine the optimal type, duration, and intensity of exercise that incorporates self-management principles to improve fatigue.

We acknowledge that exercise is not always effective for people with chronic conditions in certain situations; clients with CFS reported a worsening of fatigue after receiving graded exercise training (GET), and GET was one of the risk factors for severe illness (*p*  =  0.02).^89^ One recommendation is that if symptoms get worse after increasing the intensity or duration of exercise, clients should stay at their current level of exercise until the symptoms stabilise.^89^ This corresponds to the exercise programmes included in our review, which were inspired by self-management principles. Continuous monitoring of fatigue levels and exercise progress and adjusting exercise intensity and duration accordingly may prevent people from exacerbating their symptoms.

Because fatigue appears similar among individuals with chronic conditions,^2^ this review could provide clinicians and individuals with chronic conditions a better understanding of the various types of available fatigue self-management interventions to reduce fatigue and improve quality of life. This systematic review only included studies published in English, which may limit the findings’ generalisability to broader populations. Future reviews in English and other languages would help determine how fatigue can be understood differently in different cultures and commonly used self-management approaches.

The included trials presented a high risk of bias in general, which may limit our conclusions regarding the effectiveness of fatigue self-management. Blinding participants was the most problematic bias in this review. The difficulty of blinding participants into their intervention allocation can be explained by considering participants’ active role in self-management programmes.^13^ Furthermore, there was a low certainty of evidence in our meta-analysis results because of a serious risk of bias and inconsistency across studies. Therefore, more rigorous research is needed with a low risk of bias and less heterogeneity in the future before we can strongly recommend strategies regarding OT-/PT-led fatigue self-management for people with chronic conditions.

In this study, we did not limit our focus to studies with fatigue as the primary outcome because fatigue is a complex symptom with a close relationship to other symptoms, such as psychological issues.^3^ Thus, we decided to extract data from the primary outcome of each study if it explicitly stated its primary outcome. A fatigue outcome was included regardless of the primary or secondary outcome given the focus of the fatigue self-management programmes. In our meta-analysis of fatigue outcomes, the primary outcome of nine studies was fatigue, and four studies did not explicitly specify their primary outcome. Because it is uncertain whether those four studies evaluated fatigue as a primary outcome and calculated power estimation based on a fatigue outcome, there might be a chance of type 1 and type 2 errors in the results.^90^ We recommend that future studies explicitly specify the primary outcome and state how sample size calculations were used to estimate power based on their primary outcome.

Fatigue is a complicated symptom with different aspects, including fatigue severity, impact, and multidimensional aspects.^91^ We did not differentiate these aspects because of a lack of information about the exact differences among them.^92^ Researchers will need to work to understand and explicitly explain the differences among fatigue’s different dimensions. Only then can future reviews focus on a specific aspect of fatigue and determine the effectiveness of a fatigue self-management programme in addressing it.

In summary, although the evidence for the effectiveness of some fatigue self-management programmes is not convincing, physical exercises inspired by self-management principles may reduce fatigue symptoms, improve quality of life, and enhance other functional abilities. Further research is needed to determine the optimal type, duration, and intensity of a fatigue self-management exercise.

## Supplemental Material

sj-docx-1-chi-10.1177_17423953211039783 - Supplemental material for Fatigue self-management led by occupational therapists and/or physiotherapists for chronic conditions: A systematic review and meta-analysis Click here for additional data file. Supplemental material, sj-docx-1-chi-10.1177_17423953211039783 for Fatigue self-management led by occupational therapists and/or physiotherapists for chronic conditions: A systematic review and meta-analysis by Sungha Kim, Ying Xu, Kelly Dore, Rebecca Gewurtz, Nadine Larivière and Lori Letts in Chronic Illness
