# Supplementary material for: Fatigue self-management led by occupational therapists and/or physiotherapists for chronic conditions: A systematic review and meta-analysis
Source: Chronic Illn. 2021 Sep 13;18(3):441–57. doi: 10.1177/17423953211039783 (PMC9397391; doi:10.1177/17423953211039783)

**Appendices**

**Supplementary Table 1.** Literature searching strategy (MEDLINE)

1

exp Fatigue/

2

fatigue*.mp.

3

noncommunicable disease*.mp.

4

chronic*.mp.

5

degenerative disease*.mp.

6

exp Neurodegenerative Diseases/

7

Neurodegenerative Diseases*.mp.

8

ongoing condition*.mp.

9

ongoing health condition*.mp.

10

persistent illness*.mp.

11

persistent disease*.mp.

12

chronic patient*.mp.

13

exp Chronic Disease/

14

chronic disease*.mp.

15

chronic condition*.mp.

16

chronic illness*.mp.

17

chronically ill.mp.

18

12 or 13 or 14 or 15 or 16 or 17

19

3 or 4 or 5 or 6 or 7 or 8 or 9 or 10 or 11 or 12 or 13 or 14 or 15 or 16 or 17

20

exp Asthma/

21

asthma*.mp.

22

exp Pulmonary Disease, Chronic Obstructive/

23

chronic obstructive pulmonary disease*.mp.

24

exp Arthritis/

25

arthritis*.mp.

26

exp Arthritis, Rheumatoid/

27

rheumatoid arthritis*.mp.

28

diabetes*.mp.

29

exp Diabetes Mellitus/

30

exp Emphysema/

31

Emphysema*.mp.

32

exp Hypertension/

33

hypertension*.mp.

34

exp Myocardial Ischemia/

35

myocardial ischaemia*.mp.

36

exp Coronary Disease/

37

coronary disease*.mp.

38

congestive heart failure.mp.

39

exp Heart Failure/

40

heart failure*.mp.

41

exp Cardiovascular Diseases/

42

cardiovascular disease*.mp.

43

exp Stroke/

44

stroke*.mp.

45

exp Cerebrovascular Disorders/

46

cerebrovascular disorder*.mp.

47

cerebrovascular accident*.mp.

48

cerebrovascular accident*.mp.

49

exp Multiple Sclerosis/

50

multiple sclerosis*.mp.

51

exp Neoplasms/

52

neoplasms*.mp.

53

cancer*.mp.

54

exp Osteoporosis/

55

osteoporosis*.mp.

56

exp Fibromyalgia/

57

Fibromyalgia*.mp.

58

exp Rhinitis, Allergic, Seasonal/

59

rhinitis allergic seasonal*.mp.

60

exp HIV Infections/

61

HIV Infections*.mp.

62

exp Irritable Bowel Syndrome/

63

Irritable Bowel Syndrome*.mp.

64

exp Crohn Disease/

65

Crohn's Disease*.mp.

66

Crohn Disease*.mp.

67

exp Colitis, Ulcerative/

68

Ulcerative colitis*.mp.

69

exp Inflammatory Bowel Diseases/

70

Inflammatory Bowel Diseases*.mp.

71

exp Depression/

72

depression*.mp.

73

exp Psychotic Disorders/

74

Psychotic Disorders*.mp.

75

psychosis.mp.

76

exp Mental Disorders/

77

mental disorders*.mp.

78

mental illness*.mp.

79

exp Depressive Disorder/

80

Depressive Disorder*.mp.

81

exp Mood Disorders/

82

Mood disorder*.mp.

83

exp Schizophrenia/

84

Schizophrenia*.mp.

85

exp Anxiety Disorders/

86

Anxiety disorders*.mp.

87

exp Fatigue Syndrome, Chronic/

88

chronic fatigue syndrome*.mp.

89

3 or 4 or 5 or 6 or 7 or 8 or 9 or 10 or 11 or 12 or 13 or 14 or 15 or 16 or 17 or 18 or 19 or 20 or 21 or 22 or 23 or 24 or 25 or 26 or 27 or 28 or 29 or 30 or 31 or 32 or 33 or 34 or 35 or 36 or 37 or 38 or 39 or 40 or 41 or 42 or 43 or 44 or 45 or 46 or 47 or 48 or 49 or 50 or 51 or 52 or 53 or 54 or 55 or 56 or 57 or 58 or 59 or 60 or 61 or 62 or 63 or 64 or 65 or 66 or 67 or 68 or 69 or 70 or 71 or 72 or 73 or 74 or 75 or 76 or 77 or 78 or 79 or 80 or 81 or 82 or 83 or 84 or 85 or 86 or 87 or 88

00:03

90

exp Occupational Therapy/

91

occupational therap*.mp.

92

physiotherap*.mp.

93

physical therap*.mp.

94

90 or 91

95

92 or 93

96

90 or 91 or 92 or 93

97

exp Lupus Erythematosus, Systemic/

98

systemic lupus erythematosus*.mp.

99

lupus*.mp.

100

exp Parkinson Disease/

101

Parkinson Disease*.mp.

102

3 or 4 or 5 or 6 or 7 or 8 or 9 or 10 or 11 or 12 or 13 or 14 or 15 or 16 or 17 or 20 or 21 or 22 or 23 or 24 or 25 or 26 or 27 or 28 or 29 or 30 or 31 or 32 or 33 or 34 or 35 or 36 or 37 or 38 or 39 or 40 or 41 or 42 or 43 or 44 or 45 or 46 or 47 or 48 or 49 or 50 or 51 or 52 or 53 or 54 or 55 or 56 or 57 or 58 or 59 or 60 or 61 or 62 or 63 or 64 or 65 or 66 or 67 or 68 or 69 or 70 or 71 or 72 or 73 or 74 or 75 or 76 or 77 or 78 or 79 or 80 or 81 or 82 or 83 or 84 or 85 or 86 or 87 or 88 or 97 or 98 or 99 or 100 or 101

103

(tired* or lassitude or letharg* or weary or weariness or exhaustion or exhausted or lacklustre or ((asthenia or asthenic) adj3 syndrome) or ((lack or loss or lost) adj3 (energy or vigour))).mp.

104

1 or 2 or 103

105

94 and 102 and 104

106

95 and 102 and 104

**Supplementary Table 2.** Summary of findings

**Exercise Programmes**

| **Author**  **[Year, Country]** | **Aim** | **Design** | **Sample** | **Intervention** | **Outcome measures** | **Results** |
| --- | --- | --- | --- | --- | --- | --- |
| Yuen et al. [2007, USA] | To assess the efficacy of home based aerobic exercise and resistance exercise compared with usual care control in breast cancer population. | Three-armed randomised controlled trial | Resistance Exercise (RE) group  (n=7) Mean age: 53.7$\pm$11.3 Aerobic Exercise group (AE) group  (n=8) Mean age: 53.1$\pm$13.5  Control group  (n=7) Mean age: 55.0$\pm$13.4 | 12 Weeks Home-based AE group  -3 days a week  - Warming-up + walking + cooling-down  -Provided a PT  12 weeks Home-based RE group  -8 resistance training exercise  -No more than 3 days per week on non-consecutive days  -Delivered by a PT | Fatigue assessment: the Piper Fatigue Scale (PFS)  Functional capacity: the 6-minute walk test (6MWT) | Group difference in outcomes was not reported. The AE group showed improved fatigue (*p*=0.006) and RE group showed improved functional capacity (*p*=0.009) at 12 weeks. |
| McCullagh et al. [2008, Ireland] | To assess the effect of exercise on quality of life and fatigue in MS population. | Randomised controlled trial | Study group  (n=17; 3 male, 14 female)  Mean age: 40.5$\pm$12.68  Control group  (n=13; 3 male, 10 female)  Mean age: 33.58$\pm$6.1 | Study group:  3 months  Exercise programme  - Class & independent exercise  - 5 minutes warming-up and cooling down exercise + 10 minutes$\times$four stations with a 10 minutes break  - Maintain exertion levels between ‘fairly light to somewhat hard’  -Supported by a PT  Control group  Maintained their activity levels and visited a PT once a month. | Fatigue assessment  : MFIS  Quality of life:  the Functional Assessment of Multiple Sclerosis (FAMS)/ the Multiple Sclerosis Impact Scale-29 (MSIS-29) | The study group improved quality of life (FAMS: *p*=0.006) and fatigue (MFIS, *p*=0.02) compared with the control group at three months; the study group maintained improved exercise quality of life (FAMS: *p*=0.002) and fatigue (MFIS: *p*=0.02) at six months. |
| Durcan et al. [2014, Ireland] | To examine the effect of home exercise programme on sleep and fatigue in RA population. | Randomised controlled trial | Study group  (n=40; 10 male, 30 female)  Mean age: 61$\pm$8.0  Control group  (n=38; 18 male, 20 female)  Mean age: 59$\pm$12 | Study group: Home exercise programme  -12 weeks  -cardiovascular exercise with light to moderate intensity resistance exercise at 40-50%  -everyday range of motion exercise  -guided by PTs  Control group: advice on the benefits of exercise | Functional limitation: the Health Assessment Questionnaire (HAQ) Disability Index  Pain and stiffness with VAS  Fatigue: the Fatigue Severity Scale (FSS)  Sleep quality: the Pittsburgh Sleep Quality Index (PSQI) | Participants in the exercise group improved function (HAQ: *p*<0.001), pain (VAS: *p*=0.05), stiffness (VAS: *p*=0.05), sleep quality (PSQI, *p*=0.04), and fatigue (FSS: *p*=0.04) compared with the control group. |
| Mayo et al. [2014, Canada] | To evaluate the feasibility of a walking exercise programme for individuals experiencing cancer related fatigue. | Three-armed randomised controlled trial | STEPS programme during rehab  (n=8; 4 male, 4 female)  Mean age: 59.6$\pm$11.4  STEPS programme after rehab (n=10; 6 male, 4female) Mean age: 57.1$\pm$14.9  Rehab only  (n=8; 4 male, 4 female) Mean age: 54.4$\pm$12.2 | 8 - week STEPS programme  - An individualized walking programme according to participants’ walking status and fatigue level.  - Supervised by a PT  Usual care  -Received cancer rehabilitation, completed a daily fatigue diary, received a telephone call to check their fatigue level | Fatigue assessment: Functional Assessment of Chronic Illness Therapy Measurement System (FACIT) /Fatigue Symptom Inventory (FSI) / Fatigue Inference | Participants who participated in the STEPS programme at any time improved fatigue (Odds ratio= 3.68, 95% CI 1.05 to 12.88). |
| Voet et al. [2014, The Netherlands] | To test the effect of aerobic exercise training (AET) and CBT on chronic fatigue in FSHD population. | Three-armed randomised controlled trial | AET group  (n=20; 12 male; 8 female)Median age: 59 (21-68)  CBT group  (n=13; 8 male; 5 female)  Median age: 49 (24-69)  UC group  (n=24; 17 male; 7 female)Median age: 52 (20-79) | AET group  -3 weekly sessions of aerobic cycling exercises on Monark 827E ergometer for 16 weeks supervised exercise by a PT  CBT group  -6 possible 50 minutes modules (insufficient coping with the disease by a cognitive-behavioural therapist | Fatigue assessment:  CIS-f | Participants in the AET and CBT groups significantly improved their fatigue severity compared with the UC group at 16 weeks (*p*<0.05). Also, these significant differences maintained at 28 weeks for both AET (*p*<0.05) and CBT groups (*p*<0.05). |
| Abd El-Kader et al. [2015, Saudi Arabia] | To measure the effectiveness of aerobic exercise on fatigue in individuals with type 2 diabetes | Randomised controlled trial | Study group  (n=40; 20 male, 19 female)  Mean age: 43.62$\pm$6.17  Control group (n=40; 22 male, 17 female)  Mean age: 44.11$\pm$5.89 | Study group:  -3 sessions per week for 3 months  -Warming up + Aerobic exercise training with 60-70% of maximum heart rate + cooling down exercise  -Supervised by a PT  Control group: no intervention | Anti-inflammatory cytokines assessment: TNF-$\alpha$, IL-6, IL-8, BMI  Fatigue Assessment: Multidimensional Fatigue Inventory (MFI) | Inflammatory markers and MFI scores were significantly decreased in the study group (*p*$<$0.05) compared with the control group. |
| Arslan et al. [2016, Turkey] | To investigate the impact of a walking exercise on people with COPD | Randomised controlled trial | Study group  (n=32; 11 male, 21 female)  Mean age: 56.9$\pm$6.6  Control group (n=35; 6 male, 27 female)  Mean age: Not reported | Study group:  -8 week-walking exercising programme / 3 times per week  -Low-moderate intensity activities  - Received advice by a PT  Control group: No intervention | Fatigue assessment: COPD and Asthma Fatigue Scale (CAFS) | There was a significant difference in the mean fatigue scores between the groups for posttest (*p*=0.001) |
| Feldthusen et al. [2016, Sweden] | To evaluate the effects of person-centered physical therapy on fatigue in RA population. | Randomised controlled trial | Study group  (n=36; 4 male, 32 female)  Mean age: 54.2$\pm$8.5  Control group  (n=34; 4 male, 30 female)  Mean age: 52.7$\pm$10.9 | Study group: Person-centered physical therapy  - 12-week  - Self-care plan to manage fatigue  by tailoring health-enhancing physical activity and balancing life activities  - guided by a PT  Control group: usual care | General Fatigue: VAS (0-100) | Participants in the study group showed better improvement in fatigue at post-test (*p*=0.042) compared with the control group but not significant at follow-up (*p*=0.057). |
| Bruggeman-Everts et al. [2017, The Netherlands] | To test the effectiveness of Ambulant Activity Feedback (AAF) and guided Web-based mindfulness-based cognitive therapy (eMBCT) to psycho-education in cancer population with chronic fatigue | Three-armed randomised controlled trial | AAF  (n=62; 18 male, 44 female)  Mean age: 56.45$\pm$9.25  eMBCT  (n=55; 16 male, 39 female)  Mean age: 51.36$\pm$12.04  Unguided active control  (n=50 10 male, 40 female)  Mean age: 56.54$\pm$8.43 | AAF:  - 3 hours per week for 9 weeks period  - Home-based PT-guided intervention  eMBCT:  - 4 hours per week for 9 weeks  - A web-based psychologist-guided intervention (reading information, mindfulness exercise, recording experiences, reading and replying therapist’ feedback  Psycho-education:  - 10 minutes per week for 9 weeks  - Receiving emails (information about fatigue) | Fatigue severity: CIS-f | Participants in the AAF (*p*$<$0.001) and eMBVT (*p*=0.004) groups showed significant improvement in fatigue severity compared with the control group. Clinically relevant changes were 66% for the AAF group, 49% for the eMBCT group, and 12% for the psycho-education group. |
| Clark et al. [2017, UK) | To measure the effectiveness of graded exercise self-help (GES) plus specialist medical care (SMC) compared to SMC alone in CFS population. | Randomised controlled trial | Study group  (n=107; 19 male, 88 female)  Mean age: 38.1$\pm$11.1  Duration of illness (mon): 46 (23-114)  Control group (n=104; 25 male, 79 female)  Mean age: 38.7$\pm$12.7  Duration of illness (mon): 42 (25-99) | Study group: Guided graded exercise self help (GES)  -12 weeks  - A booklet (information about a six-step of graded exercise self-management)  - Guided by PTs and doctors  +  Specialist medical care (SMC)  - Prescriptions or advice regarding medication by specialist doctors  Control group: SMC alone | Fatigue assessment: the Chalder Fatigue Questionnaire (CFQ)  Quality of life: the Medical Outcomes Study Short Form Health Survey-36 Items (SF-36) physical function subscale | After 12 weeks, participants in the treatment group improved fatigue (CFQ, *p*<0.0001) and physical function (SF-36 physical function: *p*=0.006) compared with the comparison group. |
| Heine et al. [2017, The Netherlands] | To investigate the effectiveness of aerobic exercise on fatigue in MS population. | Randomised controlled trial | Study group  (n=43; 11 male, 32 female)  Mean age: 43.1$\pm$9.8  Control group  (n=46; 13 male, 33 female)  Mean age: 48.2$\pm$9.2 | Study group: Aerobic Exercise  -16 weeks (three times per week)  - An outpatient clinic supervised by experienced PT+ home-based exercise  Control group: Nurse -led consultation  -16 weeks (total three 45-minutes of consultations) | Fatigue assessment: CIS-f  Societal participation: IPA | Immediately after the treatment, the participants who received aerobic exercise significantly improved fatigue (CIS-f: *p*=0.014) compared with the control group, but no significant improvement was found in participation scores. |

**Energy Conservation Programmes**

| Furst et al. [1987, USA] | To evaluate the effectiveness of an energy conservation (EC) programme for adults with RA | Randomised controlled trial | Study group  (n=18; 2 male, 15 female)  Mean age: 57.4 (33-84)  Control group  (n=10; 1 male, 9 female)  Mean age: 50.8 (33-73) | Study group: EC programme  - 6 units per week / 1.5 hours each unit  - Workbook (body position, rest, activity analysis, and joint protection) + instructor’s guide.  Control group: Traditional OT | Activity of daily living: HAQ  Psychosocial adjustment to illness: the Psychosocial Adjustment to Illness (PAIS)  Knowledge: a true-false questionnaire  Disease activity: the Richey-Camp Articular Index  Pain & fatigue assessment: VAS added to HAQ and Activity Record (ACTRE) | No significant difference in any outcome measurements was reported in the study group compared with the control group after the intervention. |  |
| --- | --- | --- | --- | --- | --- | --- | --- |
| Vanage et al. [2003, USA] | To evaluate the effect of EC programme on fatigue symptoms in MS population. | Quasi-experimental study (crossover) | Study group  (n=21; 4 male, 17 female)  Mean age: 56.3$\pm$10.5  Control group  (n=16; 4 male, 12 female)  Mean age: 54.7$\pm$10.5 | EC course  - 1-hour session per week for 8 weeks  -The programme was modified based on participants’ disability level.  -Delivered by OTs and assistant | Fatigue assessment: FIS | There was a significant difference in fatigue impact between two groups in favour of the experimental group (FIS total: *p*$<$0.01) |  |
| Lamb et al. [2005, USA] | To measure the effect of self-study modules in EC education for individuals with MS. | Secondary data analysis of a randomised controlled trial by Mathiowetz et al. (2005 | Group 1: participants who attended all 6 sessions  (n=43; 7 male, 36 female)  Mean age: 48.2$\pm$10.0  Group 2: participants who received a least one module)  (n=49; 9 male, 40 female)  Mean age: 47.6$\pm$8.3 | Self-module  -Summary of the missed session, the opportunity to reflect on the session before the next session, and homework worksheets. | Fatigue impact: FIS  Quality of life: SF-36 (six subscales: role physical, general health, vitality, social functioning, role emotional, mental health)  Self-efficacy: Self-Efficacy for Performing Energy Conservation Strategies Assessment (SEA) | No significant difference was found between the two groups except for the mental health subscale of SF-36 (p=0.01~0.04) |  |
| Mathiowetz et al.  [2005,  USA] | To evaluate the effectiveness of energy conservation fatigue, quality of life, and self-efficacy in MS population. | Randomised controlled trial (crossover) | Based on intention-to-treat (ITT)  169 persons with MS; 29 male, 140 female  Mean age: 48.34$\pm$8.44  FSS score: 5.93$\pm$0.65  Disease duration: 9.47$\pm$7.44 | Study group:  6 weeks Energy conservation course  - 2 hours class/week  - Importance of rest, communication, body mechanics, ergonomic principles, environment modification, changing standards, setting priorities, activity analysis and modification, and living a balanced lifestyle.  -Provided by OTs  Control group: delayed control group | Fatigue impact: FIS  Quality of life: SF-36 (all eight subscales) | The study group significantly improved physical fatigue impact (FIS-Physical: *p*=0.0002) and social fatigue impacts (FIS-Social: *p*=0.0005) and also improved vitality (SF-36-Vitality: *p*<0.0001) compared with the control group. |  |
| Ghahari et al. [2010, Australia] | To measure the effectiveness of an online fatigue self-management programme for people with chronic neurological conditions. | Three-armed randomised controlled trial | Fatigue self-management  (n=34; 3 male, 31 female)  Mean age: 51.00$\pm$13.6  Information-only group  (n=28; 6 male, 22 female)  Mean age: 47.86$\pm$12.0  Control group  (n=33; 9 male, 24 female)  Mean age: 51.52$\pm$11.0 | Fatigue self-management  - 7 weeks online programme  - The importance of rest, communication, body mechanics, rearranging activity stations, setting priorities and standards and balancing a schedule  -Guided by OTs  Information-only  -Weekly information Via the Internet with the same content with the fatigue self-management  -Guided by OTs  Control | Fatigue assessment: FIS  Quality of life: the Personal Wellbeing Index (PWI)  Activity Participation: Australian version of Activity Card Sort | No significant difference between three groups for the primary outcome measures except for PWI (*p*=0.034) and FIS physical subscale (*p*=0.035). There was the difference between information only and control groups for PWI (*p*=0.036) and FIS physical subscale (*p*=0.030). |  |
| Finlayson et al.  [2011, USA] | To measure the effectiveness of teleconference-delivered fatigue management programme for individuals with MS | Randomised controlled trial | 181 persons with MS based on ITT  Mean age: 56$\pm$9  FSS: 5$\pm$1  Disease duration: 15$\pm$9  Symptom duration: 20$\pm$11  *Baseline group difference was not reported | Study group: Teleconference-delivered fatigue management programme  -6 weeks of group-based fatigue management programme  - Weekly 70-minute teleconference call  - Focusing on principles of fatigue management  -guided by an OT  Control group: waiting list | Fatigue assessment: FIS, FSS  Quality of life: SF-36 (all eight subscales: vitality, role emotion, mental health, social function, general health, role physical, physical function, bodily pain) | Participants in the immediate group improved all domains of fatigue impact (FIS cognitive: *p*=0.0013, FIS Physical: *p*=0.0144, FIS social: *p*=0.0021), and only role physical subscale of SF-36: *p*=0.0002) but not fatigue severity (*p*=0.2403). |  |
| García et al, [2013, UK] | To test the feasibility of an energy conservation programme for adults with MS | Pilot randomised controlled trial | Study group  (n=13; 3 male, 10 female)  Mean age: 45.85$\pm$9.93  Control group  (n=10; 4 male, 6 female)  Mean age: 52$\pm$7 | Study group: Energy conservation programme  -5 weeks energy conservation programme / One 2- hour session per week  - Introduction of energy conservation, ergonomics, goal setting, resting, scheduling, and planning, etc.  Control group: peer support group | Fatigue: FIS, FSS  Quality of life: Multiple Sclerosis Impact Scale (MSIS)-29  Self-efficacy: Multiple Sclerosis Self-efficacy Scale (MSSS)  Depression and sleep disturbance: Beck’s Depression Inventory Fast Screen (BDI-FS) & Epworth Sleepiness Scale (ESS) | There was no significant difference between groups at post-intervention. Only cognitive subscale of FIS improved at 6-week (FIS cognitive: *p*=0.01) and 3-month follow-up (FIS cognitive: *p*=0.001). |  |
| Kos et al. [2016, Belgium] | To investigate the effectiveness of a self-management occupational therapy intervention programme (SMOoth) on performance of daily activities in MS population. |  | Study group  (n=17)  Mean age: 37$\pm$8.2  Control group  (n=14)  Mean age: 44$\pm$8.9 | Study group:  3 weeks SMOoTh  - 3 individual sessions for 60-90 minutes.  - Self-efficacy in managing fatigue  -Provided by an OT  Relaxation  -Providing information about the impact of stress on fatigue and relaxing techniques  -Provided by a PT | Performance:  COPM Performance/satisfaction  Fatigue assessment: MFIS (as a secondary outcome) | No significant differences between two groups (COPM performance: *p*=0.23, COPM satisfaction *p*=0.39, MFIS total: *p*=0.27, MFIS physical: *p*=0.26, MFIS cognitive: *p*=0.48, MFIS psychosocial: *p*=0.26). |  |
| Blikman et al. [2017, The Netherlands] | To evaluate the effectiveness of energy conservation management on fatigue symptoms in MS population | Randomised controlled trial | Study group  (n=42; 8 male, 34 female)  Mean age: 47.7$\pm$11.0  Control group (n=44; 14 male, 30 female)  Mean age: 46.6$\pm$11.5 | Energy conservation management (ECM)  - 12 sessions for 4 months,  - Information of fatigue, importance of rest, balancing schedules, communication, priorities, activity analysis, and ergonomics.  - Provided by OTs  Information only  - Information about MS-related fatigue by nurses. | Fatigue assessment: CIS-f  Societal participation: the Impact on Participation and Autonomy questionnaire (IPA) | Study group did not present significant improvement on fatigue in CIS-f (*p*=0.58) and for four out of five IPA domains compared with individuals in the control group. Unfavourable results were found for the ECM group in IPA social relations (*p*=0.02). |  |
| Hersche et al. [2019, Switzerland) | To test the feasibility of an inpatient energy management education on fatigue (IEME) in people with MS compared to progressive muscle relaxation (PMR). | A single-blinded randomised controlled feasibility study | Study group  (n=24; 8 male, 16 female)  Mean age: 51.2$\pm$1,7  Control group  (n=23; 8 male, 15 female)  Mean age:  51.8$\pm$2.2 | Study group: IEME  - Face-to-face education sessions of 6.5 hours over 3 weeks delivered by an OT  - Energy management skills for better daily routine: understanding factors influencing energy level and the impact of fatigue on their daily lives and modifying their behaviours.  Control group: PMR  -To help enhance mental relaxation through reduction of muscle tension  -Six 1-hour group session of PMR over 3 weeks delivered by a PT | Fatigue assessment:  MFIS  Occupational assessment: Occupational self-assessment (OSA)  Quality of life assessment: SF-36  Self-efficacy assessment: University of Washington Self-Efficacy (UW-SES) for MS population and Self-Efficacy for Performing Energy Conservation Strategies Assessment | There was no significant difference between groups both in fatigue at T1 & T2.  There was statistically significant difference between groups in self-efficacy at follow-up (T2).  In terms of quality of life, there was statistically significant difference between groups in physical functioning both at T1 & T2  * Specific p-value was not reported. | |

**Multimodal Programmes**

| Cox [2002, UK] | To investigate the effectiveness of CBT and graded activity combination on symptoms of people with CFS. | Quasi-experimental study | Study group  (n=61; 13 male, 48 female)  Mean age: 33$\pm$10.8  Duration of illness (months): 58.9$\pm$43.4  Control group  (n=36; 6 male, 30 female)  Mean age: 37$\pm$11.4  Duration of illness (months): 65.3$\pm$42.4 | Study group: Occupational therapy inpatient intervention -Combined the principles of CBT and graded activity  -10 topics regarding management of CFS  Control group:  Waiting list | Quality of life: SF-36 (physical function)  Disease management:  Illness Management Questionnaire (IMQ)-maintaining activity/ IMQ-accommodating to illness  Fatigue assessment: Health and Fatigue Questionnaire/Perceived Fatigue Rating Scale (PFRS)-fatigue/PFRS-emotional distress | At discharge, the study group improved fatigue (*p*<0.05) and emotional distress (*p*<0.03) compared with individuals in the control group.  At 3 months follow-up, the study group presented improvement in fatigue (*p*<0.04) and illness management (*p*<0.03).  At 6 months after discharge, the study group showed improvement in physical function (*p*<0.03), fatigue (*p*=0.03), and illness management (*p*<0.03) |
| --- | --- | --- | --- | --- | --- | --- |
| van Weert et al. [2010, The Netherlands] | To compare the effect of physical training (PT) combined with CBT and PT alone on cancer related fatigue. | Three-armed randomised controlled trial | PT+CBT group  (n=76; 10 male, 66 female)  Mean age: 47.8$\pm$10.5  PT only group  (n=71; 14 male, 57 female)  Mean age: 49.9$\pm$11.3  WLC group  (n=62; 6 male, 56 female)  Mean age: 51.3$\pm$8.8 | 12 weeks PT+CBT  - Two 1-hour PT per week + one 2 hours CBT session per week  -PT modules included individual aerobic, muscle strength, and information.  -The focus of CBT to encourage participants to solve psychosocial & physical problems  -Physical training was supervised by 2 PTs and CBT was conducted by two psychologists  12 weeks PT alone  -Two 1-hour PT per week  Waiting list control group | Fatigue assessment: MFI | Compared with WLC group, PT+CBT group showed significant improvement only in physical fatigue (MFI: *p*<0.01) whereas PT group significantly improved four domains of fatigue (MFI) as follows: general fatigue (*p*<0.01), physical fatigue (*p*<0.001), mental fatigue (*p*<0.05) and reduced activation (*p*<0.05).  No significant difference in fatigue between PT+CBT and PT groups. |
| Cantarero-Villanueva et al., [2012, Spain] | To measure the effectiveness of core stability exercise and recovery myofascial release massage on fatigue in breast cancer population. | Randomised controlled trial | Study group  (n=32)  Mean age: 49$\pm$9  Control group (n=35)  Mean age: 48$\pm$9 | Study group: CUIDATE  programme  -8 week-Multimodal programme / 3 times per week for 90 minutes  - 24 hours of individual physical exercise+12 hours of the recovery phase  Control group: usual care | Fatigue assessment: Profile of Mood State (POMS)-subscale Fatigue | Multimodal programme significantly improved fatigue symptoms in the study group at post-intervention (POMS-fatigue subscale, *p* <0.05) The effect size was small at 6-month follow-up (POMS-fatigue subscale: *d*=0.38, 95% CI 0.05-0.66). |
| Zedlitz et al. [2012, The Netherlands] | To investigate the effectiveness of a treatment combining cognitive therapy (CO) with graded activity training (GRAT) on fatigue of stroke population compared with CO online. | Randomised controlled trial | Study group (n=38; 21 male, 17 female)  Mean age: 55.6$\pm$8.8  Control group (n=45; 22 male, 23 female)  Mean age: 54.8$\pm$9.1 | Study group:  12 weeks CO+GRAT  *CO*  -Once a week 2-hour sessions of CO by neurologists  - Pacing and relaxation on fatigue and psychological distress  *GRAT*  -Twice a week 2-hour sessions of GRAT by PTs  -Including walking on a treadmill, strength training, and homework assignments  Control group: CO alone | Fatigue assessment:  CIS-f and SOL-f | No significant difference between groups after the intervention for fatigue (CIS-f: *p*>0.1, SOL-f: *p*>0.1). Both groups improved significantly improved fatigue (CIS-f: *p*$<$0.001), but combining cognitive therapy with graded activity training resulted in greater improvement on fatigue. |
| Rietberg et al. [2014, The Netherlands] | To evaluate the effects of multidiscipline-ry rehab on fatigue in MS population. | Randomised controlled trial | Study group  (n=23; 9 male, 14 female)  Mean age: 45$\pm$9.9  Control group  (n=25; 8 male, 17 female)  Mean age: 47$\pm$8.6 | Study group: MDR  - 12 weeks  - Two 45 minutes sessions a week  - Promoting self-management behaviours  -Collaborated with PT, OT, and social work  MS-nurse consultation  -Received consultation every three weeks on planning activities, setting a priority, energy conservation. | Fatigue assessment: CIS-f  FSS & MFIS (as secondary outcome measures) | Participants in the study group did not significantly improve fatigue at baseline-12 weeks (CIS-f: *p*=0.39/FSS: *p*=0.47/MFIS:, *p*=0.71) and 12weeks to 24 weeks follow-up (CIS-f: *p*=0.14, /FSS: *p*=0.27/MFIS: *p*=0.78) compared with the control group. |
| Plow et al. [2019, USA] | To compare the effectiveness of telephone -delivered physical activity and fatigue self-management programmes in MS population. | A single-blinded, three-parallel-group randomised controlled trial | Physical activity (PA) group  (n=69; 14 male, 55 female)  Mean age: 51.2$\pm$9.2  Fatigue management plus (FM +)  Group  (n=70; 7 male, 63 female)  Mean age: 53.2$\pm$6.5  Contact-control social support (CC)  (n=69; 11 male, 58 female)  Mean age: 51.8$\pm$9.3 | PA-only intervention  - Three group teleconference sessions delivered by an OT + four individually tailored phone calls by a research assistant  - Education on participating in a pedometer-based walking programme, setting a goal, overcoming barriers, and self-monitoring progress  FM+ intervention  - The PA intervention+three more group teleconference sessions using an intervention of Managing Fatigue: A 6-week Course for Energy Conservation.^1^  Contact-control intervention  -Received information on MS related health topics through 6 sessions of group teleconference | Fatigue assessment:  FIS  Exercise engagement assessment:  Godin Leisure-Time Exercise Questionnaire (GLTEQ) | FM+ group presented significant improvement in physical activity (GLTEQ: *p*=0.01) and reduction in fatigue impact (*p*=0.03) compared to the CC group at posttest but not at follow-up.  PA only group showed significant improvements in physical activity (GLTEQ$:$*p*=0.04) compared to the CC group at posttest but not at follow-up.  There was no significant difference between PA only and FM+ in any outcomes. |
| Veenhuizen et al.  [2019, The Netherlands] | To evaluate the effectiveness of comprehensive self-management programme in people with neuromuscular disease | Randomised controlled trial | Study group (n=29: 8 male, 21 female)  Median age:  52 (37-63)  Disease duration  : 7 (0-41) years  Control group  (n=24, 9 male, 15 female)  Median age: 50 (41-60)  Disease duration  = 2 (0-39) | 16 weeks of comprehensive self management programme  Aerobic exercise  -guided by PT  -twice a week for the first 9 weeks / once a week for the last 7 weeks.  - 50-70% maximum heart rate  -tailored based on clients’ ability and preference  Exercise education  -3 x 60 mins for the first 3 weeks  -provided by PT  Energy Conservation  -Eight 90 mins sessions  -delivered by OT  - included education, discussion, individual goal setting, practicing activities, and homework  Implementation and relapse prevention  -10 group sessions  -by OTs/PTs or sports trainers  -encouraged implementation and explored home exercise to prevent relapse | Primary: Canadian Occupational Performance Measure (COPM)-Performance scale  Secondary: CIS-f | COPM-performance scale:  The intervention group scored higher compared to the control group both at posttest (p<0.0001) and 11 months follow-up (p<0.049).  CIS-f: There was no difference in fatigue between groups at post intervention, 3 & 11 months follow-up (p<0.81 ~ 0.53). |

**Activity Pacing Programmes**

| Murphy et al. [2010, USA] | To compare the effectiveness of a tailored activity pacing intervention and a general activity pacing intervention on fatigue and pain for individuals with OA | Randomised controlled trial | Study group  (n=17; 4 male, 13 female)  Mean age: 63.9$\pm$7.8  Control group  (n=15; 4 male, 11 female)  Mean age: 59.5$\pm$6.6 | Study group: Tailored activity pacing intervention  -2weeks / 2 one-on-one sessions  -Provided a tailed recommendation by an OT  Control group: General pacing instruction | Pain assessment:  the Western Ontario and McMaster Universities Osteoarthritis Index (WOMAC)  Fatigue assessment: the Brief Fatigue Inventory  (BFI) – severity and inference | The study group showed improvement in fatigue interference (BFI inference: *p*=0.02) compared with the control group. No significant difference in pain between groups  (WOMAC: *p*=0.35). |
| --- | --- | --- | --- | --- | --- | --- |
| Kos et al., [2015, Belguim] | To investigate the effectiveness of activity pacing self-management (APSM) on performance of daily activities in CFS population. | Randomised controlled trial | Study group  (n=16)  Mean age: 39.3$\pm$11.4  Control group  (n=17)  Mean age: 40.8$\pm$11.1 | Study group:  - 3 weeks APSM  - 60-90 minutes of individual session per week  - Daily activities within capacity  and controlling activities  - Provided by an OT  Control group: Relaxation  - PT-led Information about the impact of stress on fatigue and relaxing techniques | Performance:  COPM Performance /satisfaction | Both groups significantly improved COPM scores (*p*=0.03), but only COPM satisfaction scores were significantly different between the two groups in favour of the study group (*p*=0.02) |

**Cognitive Behavioural Therapy (CBT)**

| Stubhaug et al. [2008, Norway] | To test the effect of a comprehensive CBT programme compared with placebo-controlled mirtazapine medication in CFS population. | Three-armed randomised controlled trial  (Combined crossover and treatment-combination design) | Mirtazapine+CCBT  (n=25; 6 male, 19 female)  Mean age: 44.92$\pm$8.41  Placebo+CCBT (n=24; 3 male, 21 female)  Mean age:  44.96$\pm$8.46  CCBT+Mirtazapine  (n=11; 2 male, 9 female)  Mean age:  47.18$\pm$10.64  CCBT+Placebo  (n=12; 2 male, 10 female)  Mean age: 51.17$\pm$7.26 | Study group: CCBT  -12 weeks programme  -Two 1.5 h sessions of group therapy  - CBT by a psychiatrist and psychiatric nurse + body awareness therapy by PTs+ individual self-managed exercise including setting daily exercise goals  Control group: Medication  Mirtazapine v. inactive placebo | Fatigue: the Fatigue Scale  Clinical global impression of severity: the Clinical Global Impression dimensions of severity of illness (CGI-S) and CGI impression (CGI-I) | After 12 weeks of intervention, participants in the CCBT group improved fatigue (*p*=0.014) and clinical global impression of severity *(p*=0.001). After 24 weeks, the initial CCBT group with 12 weeks of mirtazapine significantly improved fatigue (*p*<0.001) and clinical global impression (*p*=0.002) compared with other groups |
| --- | --- | --- | --- | --- | --- | --- |
| Hewlett et al. [2011, UK] | To measure the effect of CBT for fatigue self-management in RA population compared with information alone. | Randomised controlled trial | Study group  (n=65; 16 male, 49 female)  Mean age: 61.1$\pm$10.5.  Control group  (n=62; 18 male, 44 female)  Mean age: 58.25$\pm$12.0 | CBT  - 6 $\times$ 2 hours session per week + 1 hour consolidation session at week 14  - Guided by a clinical psychologist and OT  Information only  - 1-hour didactic session  - Guided by rheumatology specialist nurse | Fatigue assessment: MAF and VAS | Participants in the CBT group improved fatigue impact (MAF: *p*=0.008, VAS: *p* <0.001) compared with the control group. |
| Hewlett et al. [2019, UK] | To compare the effectiveness of CBT plus usual care on fatigue in RA population compared with usual care alone. | 7-centre randomised controlled trial | Study group (n=156; 31 males, 125 female)  Age: median 63.7, IQR 54.2, 69.9  Control group  (n=152; 31 males, 121 female)  Age: median 61.8, IQR 54.4, 69.6 | Study group:  RAFT  - Group CBT  - Seven sessions for week 14  - Behaviours, thoughts, and feelings related to fatigue  - Delivered by rheumatology nurses/OTs  + Usual care  - The self-management booklet / nurse-led discussions  Control group: usual care alone | Fatigue impact assessment:  Bristol RA Fatigue Numerical Rating Scale (BRAF-NRS Effect) | There was significant difference between groups in favour of the study group both at 26 weeks and over two years (BRAF-NRS effect at 26 week: *p*=0.03 / BRAF-NRS effect over two years: *p*=0.01) |

**Comprehensive Fatigue Management**

| Hugos et al. [2010, USA] | To investigate the effect of a formal group fatigue programme on fatigue and self-efficacy in MS population. | Randomised controlled trial (crossover) | Study group  (n=15; 2 male, 13 female)  Mean age: 55.41$\pm$9.10  Control group  (n=15; 4 male, 11 female)  Mean age: 58.44$\pm$7.68 | Study group:  6 weeks Fatigue: Take Control  - 2 hours for DVD watching per week  - Watching 15-30 minutes of 5 DVDs highlighting the most important aspect of MS fatigue, group discussions, individual goal setting, and homework assignments  - Guided by OTs  Control group: waiting list | Fatigue assessment: MFIS  FSS (as a secondary outcome) | Participants in the study group showed significant improvement in fatigue impact (total MFIS: *p*=0.008, MFIS Physical: *p*=0.001, and MFIS Psychosocial: *p*=0.019) compared with the control group.  There was no significant difference in fatigue severity (FSS: *p*=0.468) |
| --- | --- | --- | --- | --- | --- | --- |
| Thomas et al. [2013, UK] | To measure the effectiveness of a group-based fatigue management programme (FACETS) in MS population. | Randomised controlled trial | Study group  (n=84; 23 male, 61 female)  Mean age: 48$\pm$10.2  Control group  (n=80; 22 male, 58 female)  Mean age: 50.1$\pm$9.1 | Study group: FACETS  -6 weeks group-work programme  -Up to 90 minutes session per week  -Focusing on normalization of fatigue experiences and effective use of energy  -Delivered by OTs, nurses, or PTs  CLP  -Received general advice and information about MS-fatigue by various health professionals | Fatigue severity: Global Fatigue Severity (GFS) subscale of the Fatigue Assessment Instrument (FAI)  Quality of life: MSIS-29  Self-efficacy: Multiple Sclerosis-Fatigue Self-Efficacy scale | 1 month after the final session, participants in the study group improved certainty in controlling fatigue (Multiple Sclerosis-Fatigue Self-Efficacy scale: *p*=0.001) compared with the control group. There was no significant improvement in fatigue (GFS subscale of FAI: *p*=0.86) and quality of life (MSIS-29: *p*=0.46) compared with the control group. At the second follow-up, the study group showed significant improvement in global fatigue severity (GFS subscale of the FAI: *p*=0.01) and fatigue self-efficacy (Multiple Sclerosis-Fatigue Self-Efficacy scale: *p*=0.048). |
| Pinxsterhuis et al. [2017, Norway] | To assess the effectiveness of a group-based self-management programme in CFS population. | Randomised controlled trial | Study group  (n=71; 4 male, 67 female)  Mean age: 44.0$\pm$11.8  Control group  (n=66; 12 male, 54 female)  Mean age: 43.8$\pm$11.6 | Study group: Group-based self-management programme  -16 weeks period  -8 meetings of 2.5 hours to cover topics regarding management of CFS  -Conducted by an OT and peer counselor  Control group: usual care | Quality of life:SF-36 (physical function subscale)  Fatigue assessment: FSS (as a secondary outcome) | No significant difference was reported for the primary outcome (SF-36 physical function: *p*=0.21) at six-month follow-up.  The control group showed improvement in fatigue severity (FSS: *p*=0.039), at six-month follow-up, but this significant difference did not last at one-year follow-up (FSS: *p*=0.13). |

**4-armed RCT**

| White et al. [2011, UK] | To compare the effect of adaptive pacing therapy (APT), CBT, graded exercise therapy (GET), and specialist medical care (SMC) for people with CFS. | Four-armed randomised  controlled trial | SMC  (n=160; 38 male, 122 female)  Mean age: 37$\pm$11  APT  (n=159; 38 male, 121 female)  Mean age: 39$\pm$11  CBT  (n=161; 32 male, 129 female)  Mean age: 39$\pm$12  GET  (n=160; 37 male, 123 female)  Mean age: 39$\pm$12 | -Maximum 14 sessions during the first 23 weeks and additional booster session at 36 weeks; no other sessions after it.  SMC  -A leaflet explaining the illness and at least three sessions of specialist medical treatment by doctors  -At least three sessions of SMC during the 12 months  APT  -Planning and pacing activity to reduce fatigue and achieve prioritized activities, and provide the best conditions for natural recovery  -Provided by OTs  CBT  -Changing behavioural and cognitive factors that result in exacerbation of the fatigue symptoms  -Provided by clinical psychologists and nurses  GET  -Gradual returning to physical activities; start with 30 minutes of light exercise five times per week, and then increase intensity.  -Guided by PTs and one exercise physiologist | Fatigue assessment: CFQ  Quality of life:  SF-36 physical function | At 52 weeks, participants in the CBT improved fatigue (CFQ:  *p*=0.0001) and physical function  (SF-36: *p*=0.0068); GET group also improved fatigue (CFQ: *p*=0.0003) and physical function (SF-36: *p*=0.0005) compared with the SMC alone group. However, the APT group did not show significant improvement both in fatigue (CFQ: *p*=0.38) and physical function (SF-36: *p*=0.18) compared with the SMC alone group. Thus, CBT and GET can be considered effective interventions to improve fatigue and physical function, but not APT. |
| --- | --- | --- | --- | --- | --- | --- |

**Supplementary Table 3.** A risk-of-bias of each study


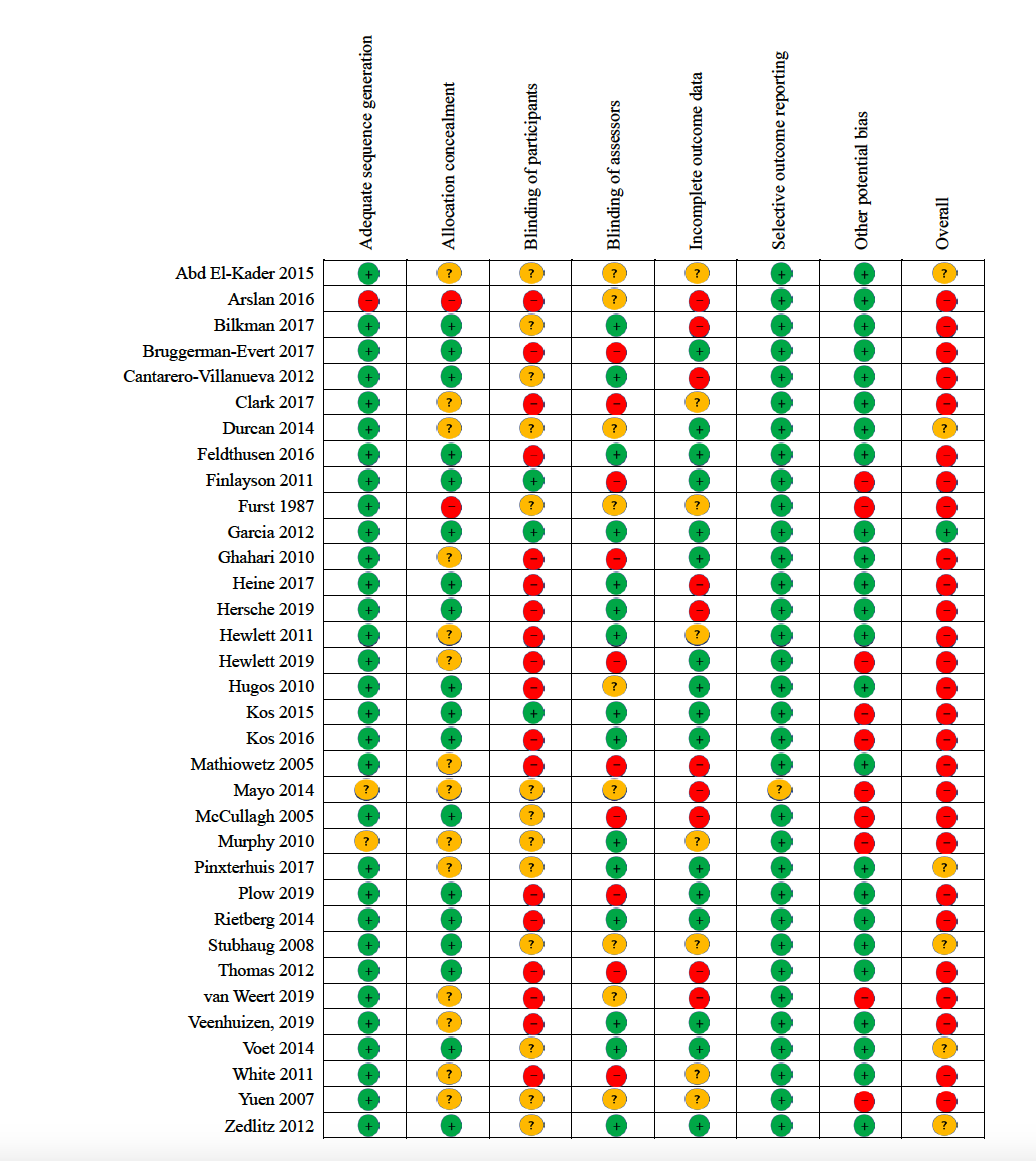


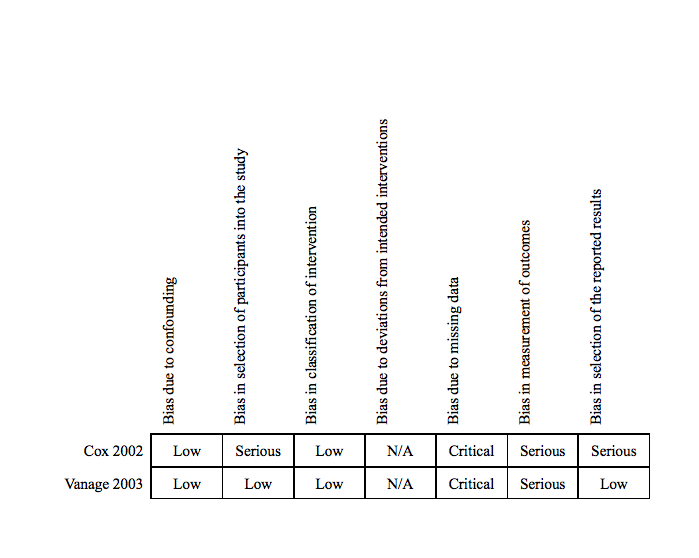

Supplement: sj-docx-1-chi-10.1177_17423953211039783 - Supplemental material for Fatigue self-management led by occupational therapists and/or physiotherapists for chronic conditions: A systematic review and meta-analysis [file sj-docx-1-chi-10.1177_17423953211039783.docx]
